# FOXD3 Is Functionally Linked to NF-κB Signaling in KRAS G12C-Mutant NSCLC Cells

**DOI:** 10.3390/cells15171564

**Published:** 2026-08-28

**Authors:** Pengzhen Xu, Jie Peng, Chao Guo, Zhu Liu, Yufeng Cao, Qing Sheng, Wenfei Xu, Xuhui Li

**Affiliations:** 1 College of Life Sciences and Medicine, Zhejiang Sci-Tech University, Hangzhou 310018, China; 2 Zhejiang Key Laboratory of Multiomics and Molecular Enzymology, Yangtze Delta Region Institute of Tsinghua University, Zhejiang, Jiaxing 314006, China; 3 Women’s Hospital, School of Medicine, Zhejiang University, Hangzhou 310018, China; 4 Qingdao Academy, Qingdao 266111, China

**Keywords:** non-small cell lung cancer, KRAS G12C, FOXD3, NF-κB signaling

## Abstract

**Highlights:**

**What are the main findings?**

- FOXD3 overexpression suppresses malignant phenotypes in two KRAS G12C-mutant NSCLC models.
- FOXD3 overexpression is associated with reduced NF-κB transcriptional activity, while TNF-α partially reverses the associated phenotypic changes.

**What are the implications of the main findings?**

- The FOXD3–NF-κB relationship represents a regulatory feature of tumor-intrinsic behavior in KRAS G12C-mutant NSCLC cells.
- Direct mechanistic and immune-competent studies are required to establish causality and potential therapeutic relevance.

**Abstract:**

KRAS G12C mutation is a clinically relevant driver in non-small cell lung cancer (NSCLC), yet the signaling networks that modulate malignant behavior in this context remain incompletely defined. In this study, we examined the functional role of FOXD3 and its relationship with NF-κB signaling in KRAS G12C-mutant NSCLC models. Stable FOXD3 overexpression was established in SW1573 and LU65 cells. FOXD3 reduced cell viability, migration, and invasion while increasing caspase 3/7 activity in both cell lines. Transcriptomic profiling in LU65 cells followed by Hallmark enrichment analysis identified TNFα signaling via NF-κB as a prominently altered pathway associated with FOXD3 overexpression. Consistently, NF-κB dual-luciferase assays showed reduced basal NF-κB transcriptional activity in FOXD3-overexpressing cells. TNFα stimulation partially reversed the inhibitory effects of FOXD3 on proliferation, migration, and invasion and attenuated FOXD3-induced apoptosis. In addition, stable FOXD3 overexpression suppressed xenograft growth in vivo. Collectively, these findings support a functional association between FOXD3 overexpression and reduced NF-κB-related transcriptional activity in KRAS G12C-mutant NSCLC models, although the present data do not establish direct causal mediation by NF-κB.

## 1. Introduction

Lung cancer continues to rank first among causes of cancer-related mortality worldwide [1]. Approximately 85% of lung cancers are classified as non-small cell lung cancer (NSCLC) [2]. Among the recurrent oncogenic events in NSCLC, KRAS activation is especially common in lung adenocarcinoma, and the G12C subtype represents one of the dominant KRAS variants, comprising roughly 39–42% of KRAS-mutant cases [3]. KRAS was long regarded as pharmacologically intractable because of its extremely high affinity for GTP and the absence of readily targetable binding pockets [4]. This concept changed after the development of allele-selective covalent inhibitors capable of binding KRAS G12C and stabilizing it in an inactive GDP-bound conformation [5]. The approvals of sotorasib and adagrasib have therefore represented an important advance in the treatment of KRAS G12C-mutant NSCLC [6,7]. Despite this progress, the magnitude and durability of clinical benefit remain constrained by adaptive and acquired resistance mechanisms [8,9,10]. These challenges highlight the need to define additional regulatory factors that shape malignant behavior in KRAS G12C-mutant NSCLC.

Beyond direct inhibition of oncogenic drivers, targeting non-oncogene dependencies has emerged as a complementary strategy for overcoming adaptive or oncogene-independent resistance. Such approaches exploit cellular processes that are not themselves oncogenic drivers but become important for tumor-cell survival under oncogenic or therapeutic stress. In NSCLC, combined targeting of oncogenic signaling and non-oncogene dependencies, including DNA-damage-response and metabolic vulnerabilities, has been proposed as a strategy to improve treatment responses and limit resistance [11]. These observations support broader investigation of regulatory factors that sustain malignant phenotypes outside the canonical KRAS signaling cascade.

NF-κB signaling has been recognized as an important regulatory axis in KRAS-driven tumor biology [12,13]. This transcriptional network contributes to several processes relevant to malignant progression, including cell survival, inflammatory signaling, epithelial–mesenchymal plasticity, and metastatic behavior [14,15]. Mutant KRAS interacts with a broad signaling network that includes MAPK/ERK, PI3K/AKT/mTOR, RalGEF/RalA/B, and NF-κB-associated inflammatory pathways [16]. Accumulating evidence indicates that NF-κB activation contributes to KRAS-mutant lung tumorigenesis, supporting this pathway as a potential therapeutic target in KRAS-driven lung cancer [17]. Identifying upstream regulators that can attenuate NF-κB signaling in the specific context of KRAS G12C mutation may therefore offer new opportunities for therapeutic intervention.

FOXD3 is a member of the forkhead box transcription factor family and plays important roles in early embryonic development and the maintenance of pluripotent cell populations [18]. FOXD3 also contributes to neural crest lineage specification and development [19]. Its role in cancer appears to be strongly context dependent. In lung cancer, previous studies have reported tumor-suppressive functions of FOXD3, including inhibition of tumor growth and angiogenesis [20] and suppression of tumor-initiating features through transcriptional repression of WDR5 [21]. Conversely, in BRAF-mutant melanoma, FOXD3 induction has been linked to adaptive resistance to RAF/MEK inhibition through upregulation of ERBB3 [22], illustrating that the biological consequences of FOXD3 activity depend on the tumor and signaling context. However, the functional relevance of FOXD3 specifically in KRAS G12C-mutant NSCLC and its relationship with NF-κB-associated signaling have not been defined. Given the established contribution of NF-κB signaling to KRAS-driven lung tumorigenesis [17,23], we therefore investigated whether FOXD3 influences malignant phenotypes and NF-κB-related transcriptional activity in this molecular context.

In the present study, we examined the biological role of FOXD3 in KRAS G12C-mutant NSCLC models and explored its relationship with NF-κB signaling. Using stable FOXD3-overexpressing SW1573 and LU65 cells, together with transcriptomic profiling, reporter assays, rescue experiments, and xenograft validation, we found that FOXD3 attenuated malignant phenotypes and was functionally associated with reduced NF-κB pathway activity. These findings support a role for FOXD3 in modulating NF-κB-associated malignant behavior in KRAS G12C-mutant NSCLC and provide a basis for further investigation of this regulatory axis in a broader lung cancer context.

## 2. Materials and Methods

### 2.1. Antibodies and Reagents

Mouse monoclonal antibodies against GAPDH (abs137959, Absin Bioscience, Shanghai, China) and FOXD3 (sc-517206, Santa Cruz Biotechnology, Santa Cruz, CA, USA) were used for Western blotting. The HRP-conjugated anti-mouse IgG secondary antibody was purchased from Cell Signaling Technology (#7076, Danvers, MA, USA). Recombinant human TNF-α was obtained from Novoprotein (Beijing, China) and dissolved in phosphate-buffered saline (PBS) before use. The NF-κB reporter plasmid (pNFκB-TA-luc), Renilla luciferase control plasmid, and Dual-Luciferase Reporter Assay System were purchased from Beyotime Biotechnology (Beijing, China). The FOXD3 overexpression construct was obtained from GeneCopoeia (Guangzhou, China).

### 2.2. Cell Lines and Culture Conditions

Human embryonic kidney 293T cells and the KRAS G12C-mutant NSCLC cell lines SW1573 and LU65 were purchased from Zhejiang Baidi Biotech (Hangzhou, China). All cell lines were authenticated by STR profiling, and the obtained profiles were compared against the Cellosaurus database, confirming their correspondence to SW1573 (RRID: CVCL_1720), LU65 (RRID: CVCL_1392), and 293T (RRID: CVCL_0063), respectively. The cells tested negative for mycoplasma and chlamydia. SW1573 cells were maintained in Leibovitz’s L-15 medium supplemented with 10% fetal bovine serum (FBS) and 100 U/mL penicillin-streptomycin. LU65 and 293T cells were cultured in RPMI-1640 medium containing 10% FBS and 100 U/mL penicillin-streptomycin. All cells were maintained at 37 °C under standard culture conditions appropriate for the corresponding medium.

### 2.3. Generation of Stable FOXD3-Overexpressing Cell Lines

Stable FOXD3-overexpressing cell lines were established using a lentiviral delivery strategy. Briefly, 293T cells were used for lentiviral packaging by co-transfection of the FOXD3 overexpression construct or the corresponding empty vector together with the packaging plasmids using Lipofectamine and PLUS reagent (Invitrogen Life Technologies, Carlsbad, CA, USA). Viral supernatants were collected at 24, 36, 48, and 60 h after transfection and stored at −80 °C in appropriate aliquots until use. SW1573 and LU65 cells were infected with the collected viral supernatants in the presence of 8 μg/mL polybrene. The viral medium was replaced with fresh medium after 12 h. At 48 h after infection, stable transductants were selected with puromycin for 7 days at 5 μg/mL for SW1573 cells and at 0.5 μg/mL for LU65 cells. The resulting stable cell populations were polyclonal pooled populations rather than single-cell-derived clones. FOXD3 overexpression was confirmed by Western blotting before functional assays, transcriptomic profiling, dual-luciferase reporter assays, TNF-α phenotypic experiments, and xenograft studies.

### 2.4. RNA Isolation and Transcriptome Sequencing

Total RNA was isolated from stable Vector and FOXD3-OE LU65 cells using the TIANGEN RNA isolation kit (DP430, Tiangen, Beijing, China) according to the manufacturer’s instructions. RNA sequencing was performed only in LU65 cells, with four independent biological replicates per group. For transcriptome analysis, RNA libraries were prepared and sequenced by Shanghai Majorbio Bio-pharm Biotechnology Co., Ltd. (Shanghai, China) on an Illumina platform (San Diego, CA, USA). Gene-level expression changes between FOXD3-OE and Vector cells were estimated using DESeq2, and log2 fold-change values were subsequently used for gene-ranking in gene set enrichment analysis.

### 2.5. Gene Set Enrichment Analysis

The Hallmark gene set collection (h.all.v7.1.symbols.gmt) was downloaded from the Molecular Signatures Database (MSigDB). Genes were ranked according to DESeq2 (version 1.40.2)-derived shrunken log2 fold-change values, and the complete ranked gene list was subjected to GSEA using the clusterProfiler package (version 4.8.2) without prior differential-expression cutoff filtering. The results were visualized with ggplot2 (version 3.4.3) as bubble plots [24,25,26,27].

### 2.6. Western Blotting

Cells were lysed in buffer containing 1% NP-40, 50 mM Tris-HCl (pH 8.0), 100 mM sodium fluoride, 30 mM sodium pyrophosphate, 2 mM sodium molybdate, 5 mM EDTA, and 2 mM sodium orthovanadate, supplemented with 10 μg/mL aprotinin, 10 μg/mL leupeptin, and 1 mM phenylmethylsulfonyl fluoride. Lysates were clarified by centrifugation at 12,000 rpm for 30 min at 4 °C, and protein concentrations were determined using a Bio-Rad protein assay kit (Bio-Rad Laboratories, Hercules, CA, USA). Equal amounts of protein were separated by SDS-PAGE and transferred to membranes for immunoblotting [28]. Membranes were incubated overnight at 4 °C with anti-FOXD3 (1:1000) or anti-GAPDH (1:3000), followed by incubation with the appropriate HRP-conjugated secondary antibody at a dilution of 1:3000 for 1 h at room temperature. Protein signals were detected using enhanced chemiluminescence reagent (Millipore, Burlington, MA, USA) and visualized with an Amersham ImageQuant 800 imaging system (Cytiva, Marlborough, MA, USA).

### 2.7. Dual-Luciferase Reporter Assay

NF-κB transcriptional activity was evaluated using a dual-luciferase reporter system. Stable Vector and FOXD3-OE SW1573 and LU65 cells were seeded into 24-well plates and co-transfected at approximately 70–80% confluence with 100 ng/well NF-κB firefly luciferase reporter plasmid and 20 ng/well Renilla luciferase control plasmid using Lipofectamine 3000 (Thermo Fisher Scientific, Waltham, MA, USA). Forty-eight hours later, cells were harvested and luciferase activity was measured according to the manufacturer’s protocol. Firefly luciferase activity was normalized to Renilla luciferase activity, and the data were expressed relative to the Vector group.

### 2.8. Cell Proliferation and Apoptosis Assays

For cell viability analysis, stable Vector and FOXD3-OE cells were seeded in 96-well plates at a density of 5 × 10^3^ cells per well. After 48 h, cell viability was assessed using the Cell Proliferation Assay Kit (Promega, Madison, WI, USA) according to the manufacturer’s instructions. For apoptosis analysis, cells were cultured under the same conditions and caspase 3/7 activity was determined using the Caspase-Glo 3/7 Assay Kit (Promega, Madison, WI, USA). For TNF-α experiments, Vector and FOXD3-OE cells were treated with recombinant human TNF-α at a final concentration of 10 ng/mL, and cell viability and caspase 3/7 activity were assessed 48 h after treatment.

### 2.9. Wound Healing Assay

Stable Vector and FOXD3-OE cells were plated in six-well plates at 1.2 × 10^6^ cells per well. After the cells reached approximately 90–95% confluence, a linear wound was generated with a 200 μL pipette tip. The cultures were then maintained in medium containing 1% FBS to minimize the contribution of cell proliferation to wound closure. Images were collected at 0 h and 36 h using a Nikon Eclipse Ti2 microscope (Nikon, Tokyo, Japan). Relative wound closure was quantified based on the change in wound width over time. For TNF-α experiments, TNF-α (10 ng/mL) was added immediately after scratching, and wound closure was evaluated at 36 h.

### 2.10. Matrigel Invasion Assay

Cell invasion was examined using Matrigel-coated Transwell chambers (Corning, NY, USA). Briefly, 5000 cells suspended in medium containing 1% FBS were added to the upper chamber, while medium supplemented with 15% FBS was placed in the lower chamber as a chemoattractant. After 48 h of incubation, non-invading cells on the upper surface were gently removed with a cotton swab. Cells that had migrated through the membrane were fixed with 4% formaldehyde for 15 min and stained with 1% crystal violet for 10 min. After washing with PBS, invading cells were counted in five randomly selected non-overlapping fields using a 20× objective. For TNF-α experiments, TNF-α (10 ng/mL) was added at cell seeding, and invasion was assessed after 48 h.

### 2.11. Xenograft Model

Female athymic nude mice (6–8 weeks old) were maintained under specific pathogen-free conditions. Mice were randomly allocated to the Vector and FOXD3-OE groups (*n* = 5 per group). A total of 3 × 10^6^ stable Vector or FOXD3-OE SW1573 cells suspended in PBS were injected subcutaneously into the right flank of each mouse. Tumor length (L) and the perpendicular width (W) were measured every 2 days beginning on day 7 after inoculation, and tumor volume was calculated as V = (L × W^2^)/2. The mice were sacrificed 19 days after inoculation. Tumors were excised, photographed, weighed, and used for subsequent analysis. All animal procedures were approved by the Laboratory Animal Welfare and Ethics Committee of Hangzhou Normal University (Approval No.: HSD-20250818-04) and were carried out in accordance with institutional guidelines.

### 2.12. CCLE/DepMap Analysis

Publicly available CCLE/DepMap transcriptomic and somatic mutation data were analyzed to assess endogenous FOXD3 expression in NSCLC cell lines. FOXD3 expression was evaluated across 143 NSCLC cell lines and separately across 13 KRAS G12C-mutant NSCLC models and is reported as log2(TPM + 1).

### 2.13. Statistical Analysis

Statistical analyses were performed using GraphPad Prism 8.0.1. Data are presented as mean ± SD unless otherwise indicated. Comparisons between two groups were analyzed using two-tailed unpaired Student’s *t*-tests, whereas comparisons among three or more groups were performed using unpaired one-way ANOVA followed by Tukey’s post hoc test. A value of *p* < 0.05 was considered statistically significant.

## 3. Results

### 3.1. FOXD3 Overexpression Suppresses Malignant Phenotypes in KRAS G12C-Mutant NSCLC Cells

To further characterize baseline FOXD3 expression in the two experimental models, we analyzed publicly available CCLE/DepMap RNA-seq data. FOXD3 expression was very low in both SW1573 and LU65 cells, with expression values of 0.0000 and 0.01375 log2(TPM + 1), respectively. Among 143 NSCLC cell lines, SW1573 and LU65 ranked at approximately the 3.87th and 11.27th percentiles of the FOXD3 expression distribution, respectively, indicating that both models lie within the low-expression range (Figure S1A). However, FOXD3 expression varied across the 13 KRAS G12C-mutant NSCLC models examined (Figure S1B), indicating that very low FOXD3 expression is not universal within this molecular subgroup. Thus, SW1573 and LU65 represent KRAS G12C-mutant NSCLC models with relatively low endogenous FOXD3 expression.

**Figure 1:**
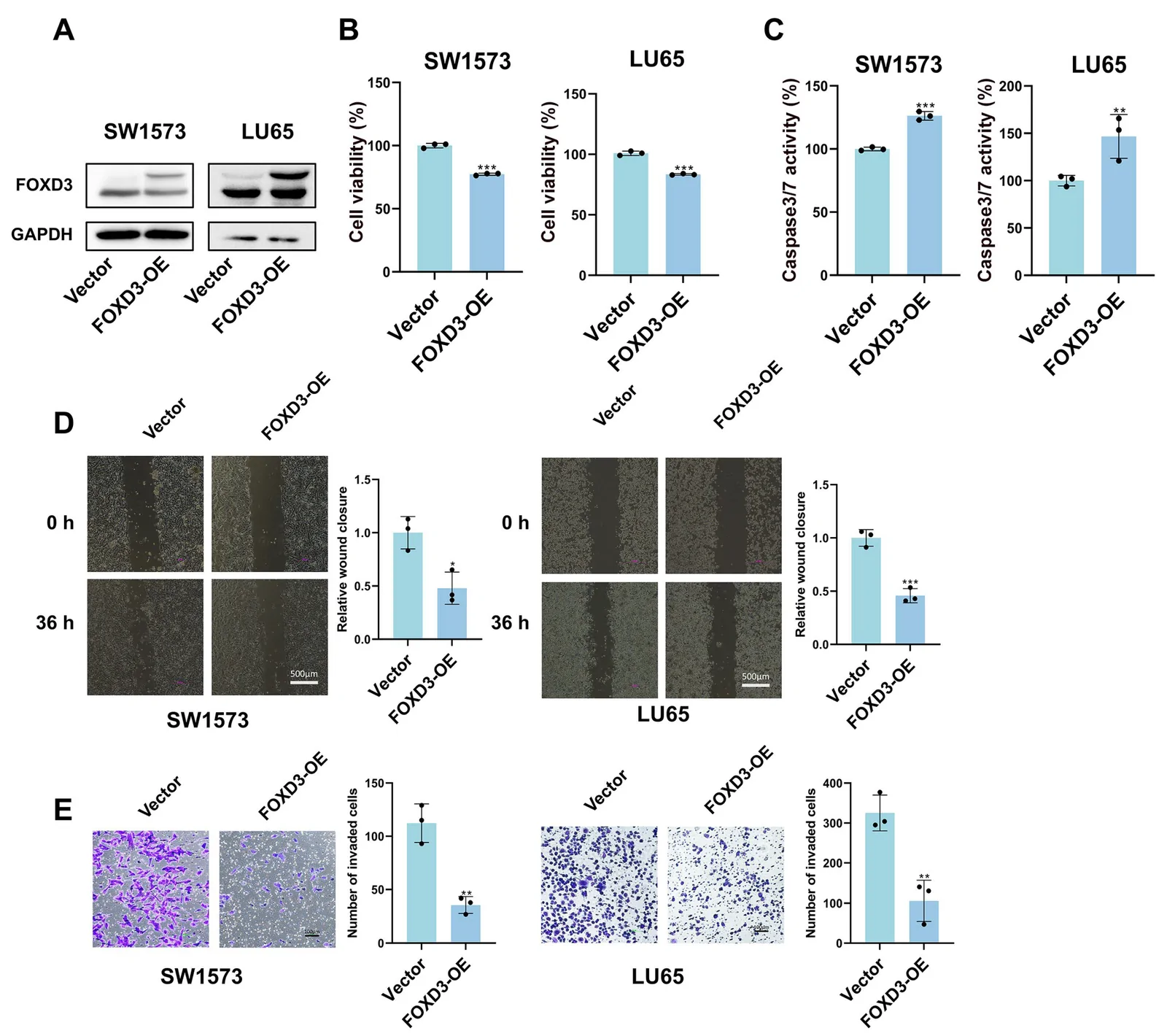
Stable FOXD3 overexpression suppresses malignant phenotypes in KRAS G12C-mutant NSCLC cells. (**A**) Western blot analysis confirming FOXD3 overexpression in SW1573 and LU65 cells with stable FOXD3 overexpression (FOXD3-OE) and corresponding vector controls (Vector). (**B**) Cell viability assay showing that stable FOXD3 overexpression significantly inhibited the proliferation of SW1573 and LU65 cells. Data are presented as mean ± SD (*n* = 3). *** *p* < 0.001. (**C**) Caspase 3/7 activity assay showing increased apoptosis in FOXD3-OE cells compared with Vector cells. Data are presented as mean ± SD (*n* = 3). ** *p* < 0.01, *** *p* < 0.001. (**D**) Wound-healing assay showing that stable FOXD3 overexpression reduced the migratory capacity of SW1573 and LU65 cells. Representative images were obtained at the indicated time points. Scale bar = 500 μm. Data are presented as mean ± SD (*n* = 3). * *p* < 0.05, *** *p* < 0.001. (**E**) Transwell invasion assay showing that stable FOXD3 overexpression significantly impaired the invasive ability of SW1573 and LU65 cells. Representative images and corresponding quantitative analysis are shown. Scale bar = 100 μm. Data are presented as mean ± SD (*n* = 3). ** *p* < 0.01.

To investigate the functional role of FOXD3, we established FOXD3-overexpressing cell models in two KRAS G12C-mutant NSCLC cell lines, SW1573 and LU65. Ectopic FOXD3 expression was confirmed by Western blotting (Figure 1A). Cell viability assays showed that FOXD3 overexpression significantly suppressed proliferation in both cell lines (Figure 1B). In addition, caspase-3/7 activity assays demonstrated that FOXD3 overexpression promoted apoptosis in SW1573 and LU65 cells (Figure 1C). Wound healing and Matrigel invasion assays further showed that FOXD3 overexpression markedly reduced the migratory and invasive capacities of both cell lines (Figure 1D,E).

### 3.2. FOXD3 Is Associated with Reduced NF-κB Pathway Activity in KRAS G12C-Mutant NSCLC Models

**Figure 2:**
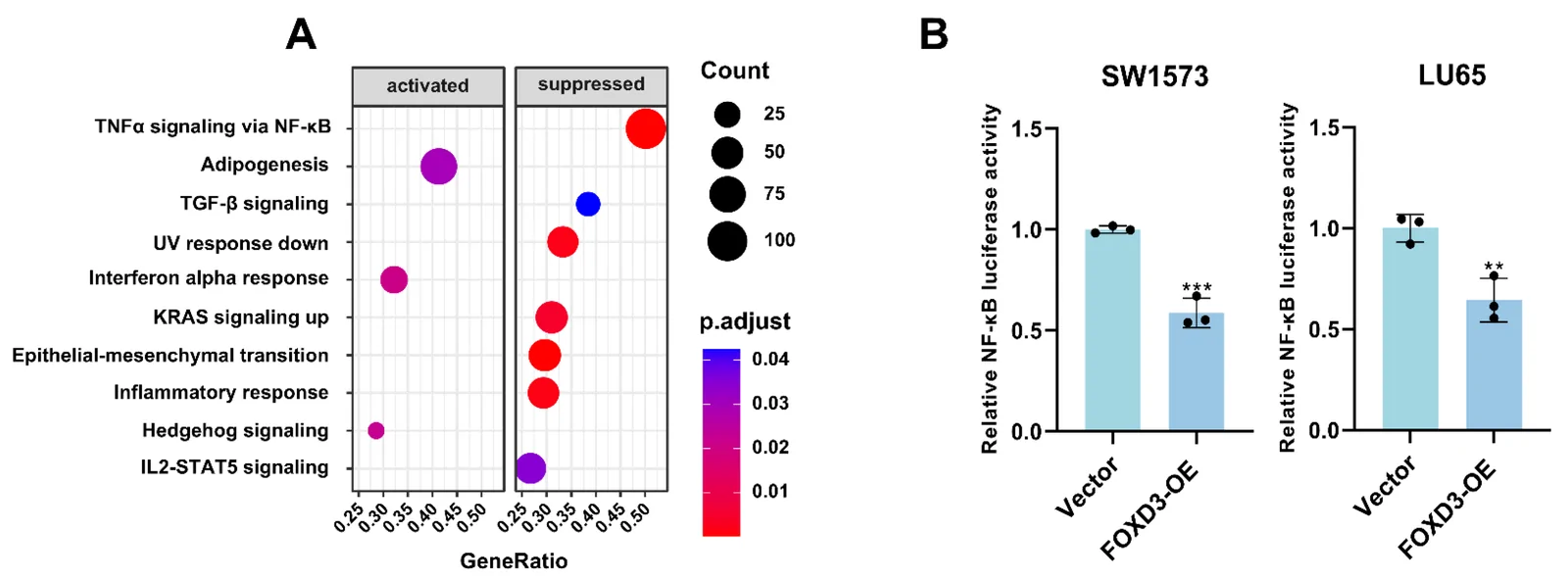
Transcriptomic and reporter analyses implicate NF-κB signaling as a pathway associated with FOXD3 activity in KRAS G12C-mutant NSCLC cells. (**A**) Hallmark gene set enrichment analysis of the ranked transcriptomic profile from stable Vector and FOXD3-OE LU65 cells. Enriched gene sets in each group are displayed. Dot size denotes gene count, the x-axis represents GeneRatio, and color indicates the adjusted *p* value. TNFα signaling via NF-κB was prominently enriched in Vector cells compared with FOXD3-OE cells. (**B**) Dual-luciferase assay showing reduced basal NF-κB transcriptional activity in FOXD3-OE SW1573 and LU65 cells relative to Vector controls. Data are presented as mean ± SD (*n* = 3). ** *p* < 0.01, *** *p* < 0.001.

To explore the downstream signaling pathways regulated by FOXD3, RNA sequencing was performed in LU65 cells with stable FOXD3 overexpression. Hallmark enrichment analysis showed that TNFα signaling via NF-κB was among the top pathways enriched in Vector cells compared with FOXD3-OE cells (Figure 2A), suggesting an association between FOXD3 overexpression and reduced NF-κB-related transcriptional programs. Consistent with this observation, NF-κB reporter assays showed that FOXD3 overexpression reduced basal NF-κB transcriptional activity in both SW1573 and LU65 cells (Figure 2B), supporting a functional association between FOXD3 and NF-κB pathway suppression.

### 3.3. TNF-α Stimulation Partially Reverses the Phenotypic Effects Associated with FOXD3 Overexpression

**Figure 3:**
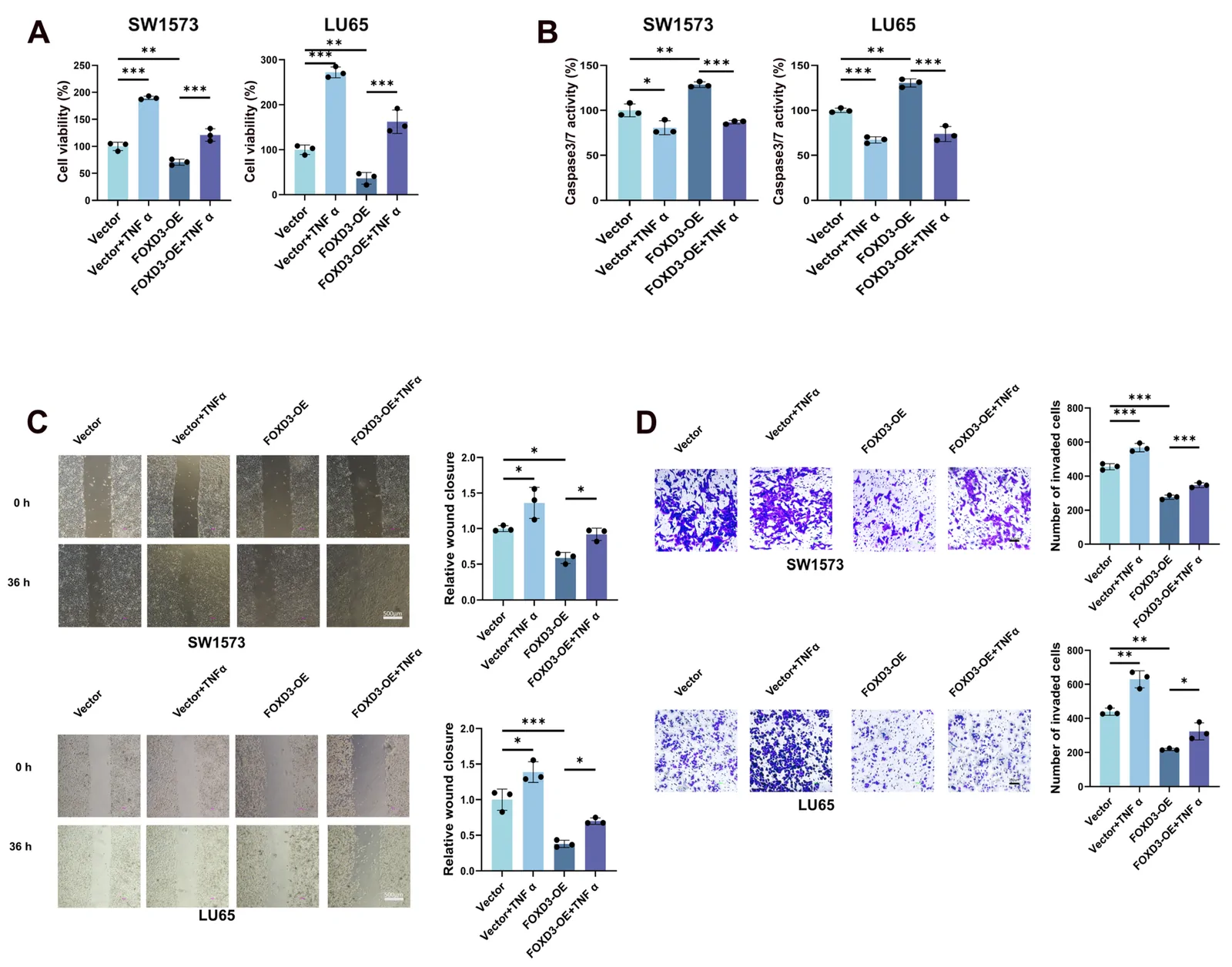
TNFα stimulation partially reverses the phenotypic effects of FOXD3 overexpression in KRAS G12C-mutant NSCLC cells. (**A**) Cell viability assay showing that TNFα treatment partially restored the proliferative capacity of FOXD3-OE SW1573 and LU65 cells. Data are presented as mean ± SD (*n* = 3). ** *p* < 0.01, *** *p* < 0.001. (**B**) Caspase 3/7 activity assay showing that TNFα treatment attenuated FOXD3-induced apoptosis in SW1573 and LU65 cells. Data are presented as mean ± SD (*n* = 3). * *p* < 0.05, ** *p* < 0.01, *** *p* < 0.001. (**C**) Wound-healing assay showing that TNFα treatment partially reversed the inhibitory effect of stable FOXD3 overexpression on cell migration. Representative images were obtained at 0 and 36 h, and the relative wound closure was quantified. Scale bar = 500 μm. Data are presented as mean ± SD (*n* = 3). * *p* < 0.05, *** *p* < 0.001. (**D**) Transwell invasion assay showing that TNFα treatment partially restored the invasive ability of FOXD3-OE SW1573 and LU65 cells. Representative images and corresponding quantitative analysis are shown. Scale bar = 100 μm. TNFα, tumor necrosis factor alpha. Data are presented as mean ± SD (*n* = 3). * *p* < 0.05, ** *p* < 0.01, *** *p* < 0.001.

Based on the transcriptomic and reporter evidence implicating NF-κB signaling as a downstream pathway of FOXD3, we next performed rescue experiments using TNFα stimulation in SW1573 and LU65 cells. TNFα treatment partially restored cell viability in FOXD3-OE cells while attenuating FOXD3-induced caspase 3/7 activity (Figure 3A,B). In addition, TNFα partially reversed the inhibitory effects of FOXD3 overexpression on migration and invasion, as demonstrated by wound-healing and Transwell invasion assays (Figure 3C,D). These findings show that TNF-α stimulation partially reverses the phenotypic changes associated with FOXD3 overexpression. However, because TNF-α can engage multiple downstream signaling processes, these experiments do not establish that NF-κB directly mediates the phenotypic effects of FOXD3.

### 3.4. FOXD3 Suppresses Tumor Growth In Vivo

**Figure 4:**
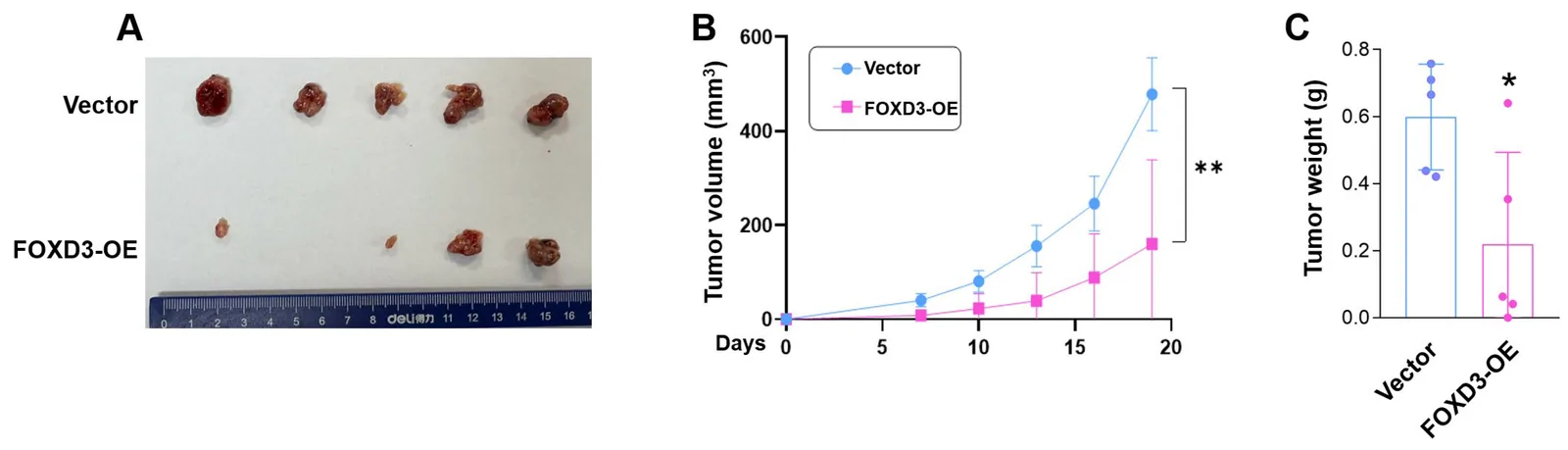
FOXD3 overexpression suppresses tumor growth in vivo. (**A**) Representative images of xenograft tumors excised from mice subcutaneously injected with Vector or FOXD3-OE SW1573 cells. (**B**) Tumor growth curves showing that stable FOXD3 overexpression significantly inhibited xenograft growth over time. Data are presented as mean ± SD (*n* = 5 mice per group). ** *p* < 0.01. (**C**) Final tumor weights measured at the experimental endpoint. Data are presented as mean ± SD (*n* = 5 mice per group). * *p* < 0.05.

Given the inhibitory effects of FOXD3 on malignant phenotypes in vitro, we next assessed whether FOXD3 also affects tumor growth in vivo. Stable Vector or FOXD3-OE SW1573 cells were subcutaneously implanted into nude mice. Xenografts derived from FOXD3-OE cells were visibly smaller than those formed by Vector cells at the endpoint (Figure 4A). Consistently, tumor growth curve analysis showed that FOXD3 overexpression significantly suppressed xenograft growth over time (Figure 4B). Moreover, the final tumor weights were significantly lower in the FOXD3-OE group than in the Vector group (Figure 4C). These findings further support a tumor-suppressive role of FOXD3 in vivo.

## 4. Discussion

KRAS G12C has emerged as a therapeutically actionable alteration in NSCLC, although the durability of direct KRAS G12C-targeted treatment remains limited by adaptive and acquired resistance [8,9,10]. In the present study, we investigated FOXD3 in KRAS G12C-mutant NSCLC models and found that its overexpression was associated with attenuated malignant phenotypes and reduced NF-κB signaling activity. Rather than establishing FOXD3 as a newly identified suppressor in lung cancer, our data support a functional link between FOXD3 and NF-κB-associated signaling in KRAS G12C-mutant NSCLC models.

FOXD3 is a member of the forkhead box transcription factor family and is known to participate in embryonic development and lineage determination, whereas its roles in tumor biology appear to vary across contexts [18]. Its contribution to KRAS G12C-mutant NSCLC, however, has not been well defined. Previous studies have described tumor-suppressive effects of FOXD3 in NSCLC models outside the KRAS G12C context [20], whereas its functional relevance in KRAS G12C-mutant NSCLC has remained unclear. In our models, FOXD3 overexpression consistently impaired proliferation, migration, invasion, and cell survival in two independent KRAS G12C-mutant cell lines. The in vivo xenograft data further supported these observations by showing a marked reduction in tumor growth following FOXD3 overexpression. Collectively, these findings indicate that FOXD3 exerts suppressive effects on malignant progression in this molecular setting.

At the pathway level, transcriptomic and reporter analyses implicated NF-κB-associated transcriptional programs as being altered following FOXD3 overexpression. NF-κB signaling has been shown to play an important functional role in KRAS-driven lung tumorigenesis, and genetic suppression of NF-κB components reduces KRAS-induced lung tumor development [12,17,23]. In the present study, FOXD3 overexpression reduced basal NF-κB transcriptional activity in both SW1573 and LU65 cells, consistent with the Hallmark GSEA results showing enrichment of the TNFα signaling via NF-κB gene set in vector cells relative to FOXD3-OE cells. TNFα stimulation also partially reversed the phenotypic changes associated with FOXD3 overexpression. TNFα is a well-established activator of canonical NF-κB signaling [29,30], but it is a pleiotropic cytokine that can also engage signaling pathways beyond NF-κB, including ERK and p38 signaling [31]. Therefore, the TNFα experiments provide functional support for the involvement of TNFα/NF-κB-related signaling but do not establish NF-κB as the direct or exclusive mediator of the FOXD3-associated phenotypes. Direct manipulation of core NF-κB components, such as RELA/p65 or IKKβ, will be required to establish the causal relationship between FOXD3 and NF-κB signaling.

These observations may have potential relevance to therapeutic response in KRAS G12C-mutant NSCLC. Increasing evidence suggests that bypass signaling and inflammatory programs can reduce the durability of response to direct KRAS G12C blockade [32,33]. Beyond direct oncogene inhibition, the concept of non-oncogene addiction suggests that tumor cells may also depend on cellular stress-response, DNA-damage-response, or metabolic programs that can provide complementary therapeutic vulnerabilities. Consistent with this concept, Amato et al. demonstrated that targeting non-oncogene dependencies may improve therapeutic responses in EGFR- and KRAS-mutant NSCLC [11]. In this context, the FOXD3–NF-κB relationship identified here may warrant further evaluation in treatment-response settings. However, the present study did not directly assess sensitivity to KRAS G12C inhibitors or resistance models. Therefore, any implication for therapeutic resistance should be considered preliminary and will require dedicated validation in future studies.

Several limitations should be considered. First, the present study relied on FOXD3 overexpression, and complementary loss-of-function experiments in KRAS G12C-mutant cells with higher endogenous FOXD3 expression would strengthen the causal evidence for endogenous FOXD3 function. Second, although the transcriptomic and reporter data, together with the TNFα phenotypic experiments, support a functional association between FOXD3 and NF-κB-related signaling, TNFα is pleiotropic and the study did not directly manipulate core NF-κB components such as RELA/p65 or IKKβ. Therefore, direct causal mediation by NF-κB remains to be established. Third, RNA sequencing was performed only in LU65 cells, whereas the xenograft study was performed only with SW1573 cells, limiting the generalizability of these observations across KRAS G12C-mutant NSCLC models. Fourth, the xenograft experiments used athymic nude mice, which lack functional T cells. Although this model enables evaluation of tumor-intrinsic effects, it cannot fully capture the contribution of the intact immune microenvironment, which is particularly relevant to TNFα/NF-κB signaling. Future studies using immunocompetent syngeneic or genetically engineered KRAS-mutant lung cancer models will be required to address this question. Finally, the precise molecular mechanism linking FOXD3 to altered NF-κB transcriptional activity remains unresolved and will require direct biochemical and promoter-level analyses.

## 5. Conclusions

FOXD3 overexpression attenuated malignant phenotypes in KRAS G12C-mutant NSCLC cells and suppressed SW1573 xenograft growth in vivo. Transcriptomic and reporter analyses support a functional association between FOXD3 overexpression and reduced NF-κB-related transcriptional activity, while TNFα stimulation partially reversed the associated in vitro phenotypes. These findings identify a FOXD3–NF-κB-related regulatory relationship in KRAS G12C-mutant NSCLC models, but direct causal mechanisms and their therapeutic relevance require further investigation.

## Data Availability

The RNA-sequencing data generated in this study have been deposited in the SRA repository under accession number [BioProject: PRJNA1517737]. The other data supporting the findings of this study are available within the article and its Supplementary Materials. Additional data are available from the corresponding author upon reasonable request.

## Supplementary Materials

The following supporting information can be downloaded at: https://www.mdpi.com/article/10.3390/cells15171564/s1, Figure S1: FOXD3 expression in NSCLC cell lines in the CCLE/DepMap dataset.
