# Supplementary material for: FOXD3 Is Functionally Linked to NF-κB Signaling in KRAS G12C-Mutant NSCLC Cells
Source: Cells. 2026 Aug 28;15(17):1564. doi: 10.3390/cells15171564 (PMC13564895; doi:10.3390/cells15171564)
Supplement: Supplementary file 1 [file cells-15-01564-s001.zip › cells-4496376-supplementary.pdf]

Supplementary Figure

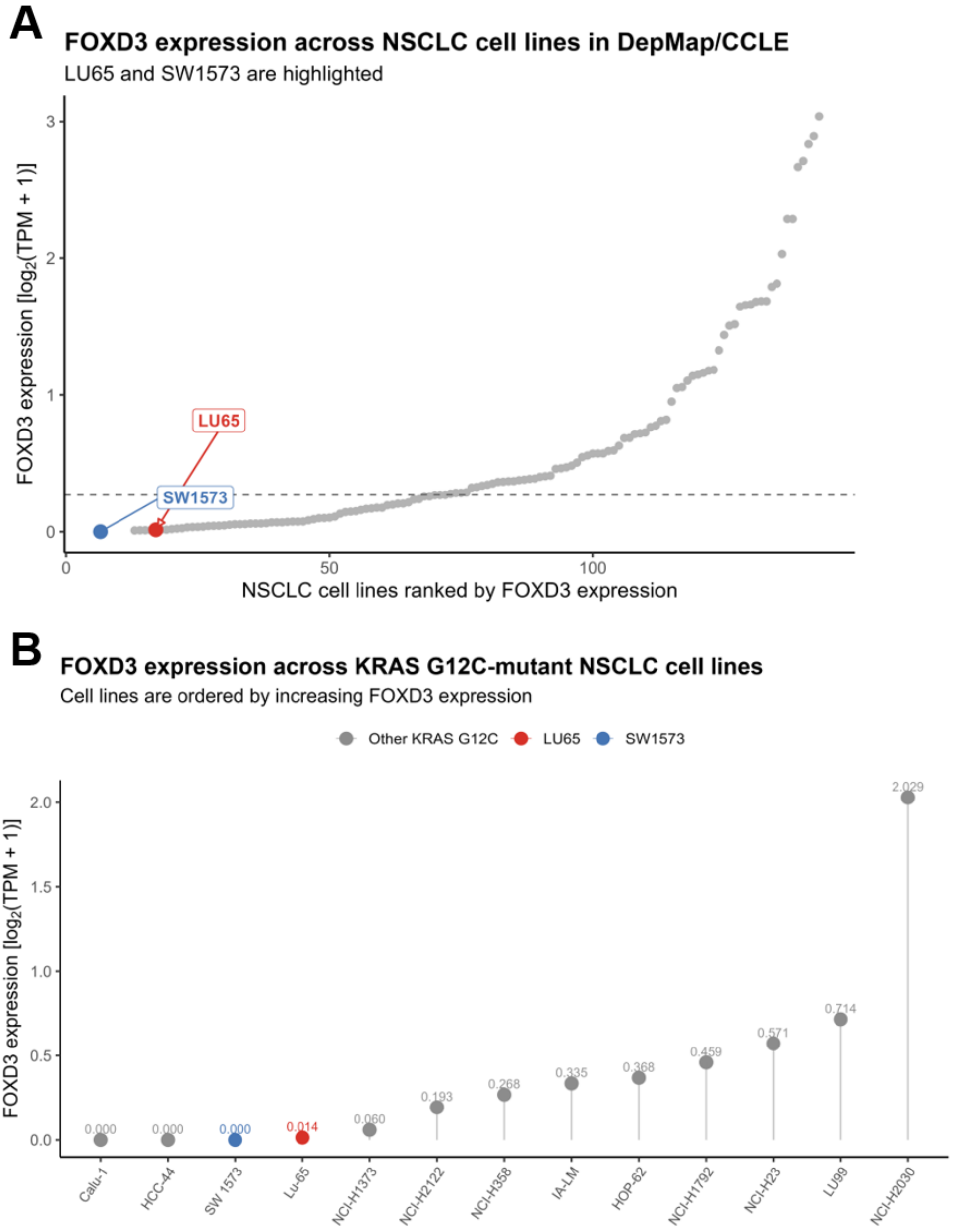

Figure S1. FOXD3 expression in NSCLC cell lines in the CCLE/DepMap dataset. (A) FOXD3 expression across 143 NSCLC cell lines. SW1573 and LU65 are highlighted and ranked at approximately the 3.87th and 11.27th percentiles of the overall FOXD3 expression distribution, respectively. (B) FOXD3 expression across KRAS G12C-mutant NSCLC cell lines. FOXD3 expression varied across KRAS G12C-mutant models, indicating that low FOXD3 expression is not universal within this molecular subgroup. SW1573 and LU65 are highlighted. FOXD3 expression is shown as log2(TPM+1) based on CCLE/DepMap RNA-seq data.
